# Insights into how environment shapes *post-mortem* RNA transcription in mouse brain

**DOI:** 10.1038/s41598-021-92268-y

**Published:** 2021-06-21

**Authors:** Raphael Severino Bonadio, Larissa Barbosa Nunes, Patricia Natália S. Moretti, Juliana Forte Mazzeu, Stefano Cagnin, Aline Pic-Taylor, Silviene Fabiana de Oliveira

**Affiliations:** 1 Department of Genetics and Morphology, University of Brasilia, Brasilia, Brazil; 2 Faculty of Medicine, University of Brasilia, Brasilia, Brazil; 3 Department of Biology and CRIBI Biotechnology Centre, University of Padova, Padova, Italy

**Keywords:** Gene expression, Genetic markers

## Abstract

Most biological features that occur on the body after death were already deciphered by traditional medicine. However, the molecular mechanisms triggered in the cellular microenvironment are not fully comprehended yet. Previous studies reported gene expression alterations in the post-mortem condition, but little is known about how the environment could influence RNA degradation and transcriptional regulation. In this work, we analysed the transcriptome of mouse brain after death under three concealment simulations (air exposed, buried, and submerged). Our analyses identified 2,103 genes differentially expressed in all tested groups 48 h after death. Moreover, we identified 111 commonly upregulated and 497 commonly downregulated genes in mice from the concealment simulations. The gene functions shared by the individuals from the tested environments were associated with RNA homeostasis, inflammation, developmental processes, cell communication, cell proliferation, and lipid metabolism. Regarding the altered biological processes, we identified that the macroautophagy process was enriched in the upregulated genes and lipid metabolism was enriched in the downregulated genes. On the other hand, we also described a list of biomarkers associated with the submerged and buried groups, indicating that these environments can influence the post-mortem RNA abundance in its particular way.

## Introduction

Life does not end when the body dies. At least, not immediately. Intracellular biomolecules use the last energetic resources to maintain their function, even after an organism’s death. The investigation of post-mortem RNA dynamics can provide novel insights into the biology of death. Previous studies have suggested that there is some level of active post-mortem mRNA transcription^1–4^. Although most of the transcriptome is shutting down or degrading, approximately 1% of the brain transcriptome is turned on in response to death^2^. Moreover, most of the proteome follow the degradation trend, but specific proteins show a distinctive pattern along the post-mortem period. In fact, some biomolecules (e.g., caspases) can be activated even during the post-mortem period, showing that death is a dynamic process^5^. RNA degradation and synthesis after death has been investigated in the past^2,3,6^. However, most of the available data refer to studies that aimed to unravel molecular mechanisms of mental disorders, such as schizophrenia, Alzheimer and drug addiction^7–9^ and little is known about how these processes occur in healthy individuals.

Every biological system interacts with the surrounding environment, both during life and after death. Hence, there is consensus that the environment can influence mRNA transcription dynamics, mostly through epigenetic regulators^10,11^. The degradation rate of mRNAs is influenced by intrinsic factors, such as gender, age and health conditions during the antemortem period; and also external factors, such as temperature, humidity, sunlight exposure and microbiota interaction in the post-mortem period^12,13^. Therefore, it is reasonable to think that mRNA degradation and gene expression could be altered depending on the circumstances in which the cadaver was deposited. Furthermore, each tissue presents unique characteristics and different post-mortem RNA synthesis rates and degradation^14^. The degradation rate of post-mortem RNA was previously evaluated in several tissues and it was demonstrated that the brain presents higher RNA stability than the other tissues tested^15–18^.

In the last few years, mRNA degradation has been studied to solve forensic questions, such as determining the nature of biological fluids found in crime scenes, investigation of the cause of death and estimation of post-mortem interval (PMI)^19^. To overcome ethical problems in the management of post-mortem human samples, and because of the impossibility to simulate with them different concealment situations, here we performed a pilot study on brains of healthy mice, to understand the environmental effects on RNA expression without the interference of a pathological status. In this work we simulated three body concealment situations: air exposed, buried, and submerged. Our major aims were: (i) to better understand the impact of external factors in RNA expression and degradation; (ii) identify common transcripts and molecular pathways shared by concealment groups and (iii) identify exclusive transcripts, molecular pathways and list biomarkers associated to each environment.

## Results

### RNA integrity and gene expression analysis

**Figure 1:**
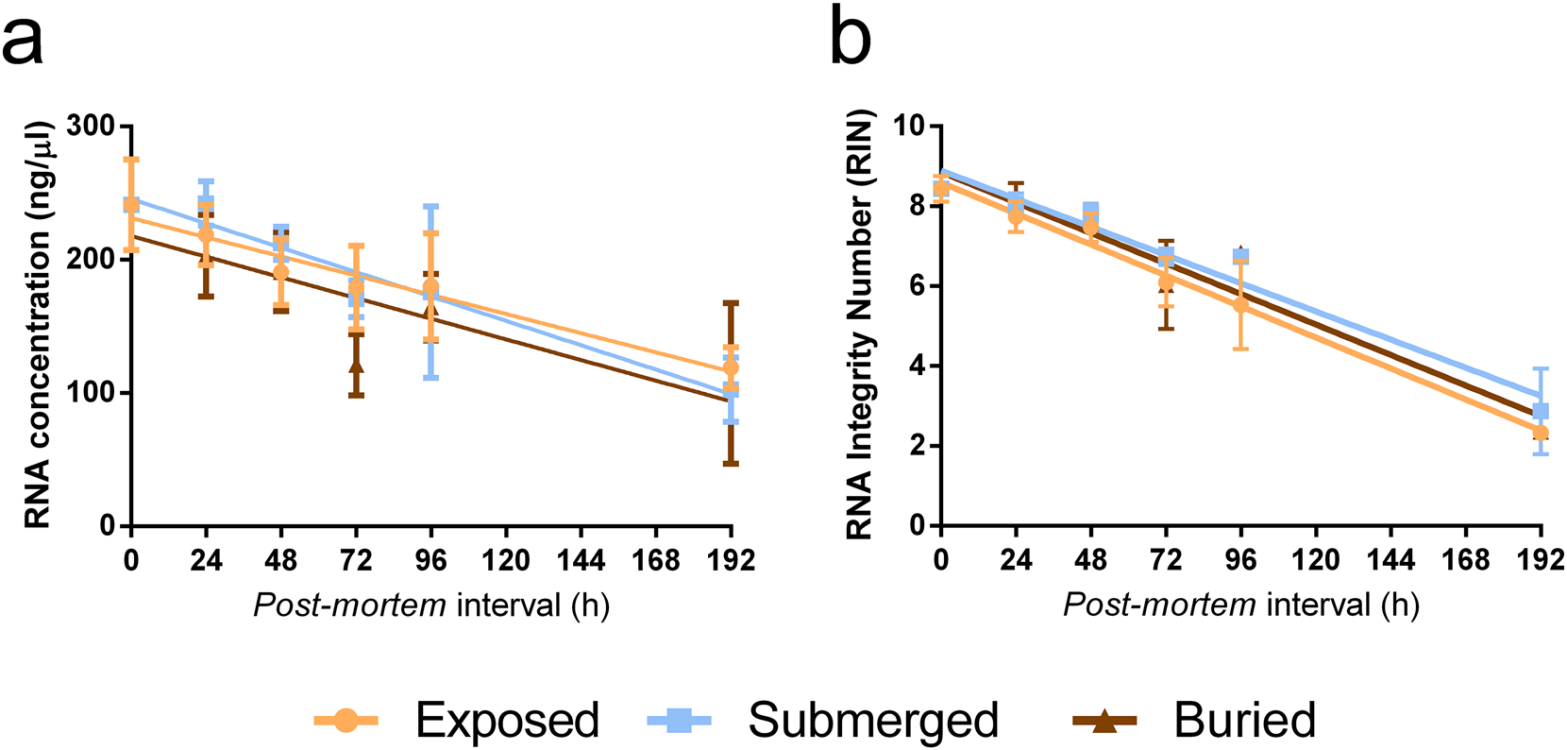
Similar degradation rate in different concealment simulations. The RNA quantification analysis (**A**) and RNA integrity analysis (**B**) show that the overall sample quantity (ng/µl) and quality (RIN) decreases over time, but at the same rate in all groups tested. The slope comparison showed no statistical differences among groups both in RNA concentration (*P* = 0.6409) and integrity (*P* = 0.644).

In this work, we analysed the RNA stability and expression in the brain of mouse. In order to test if the concealment simulations influenced the RNA concentration and integrity in our samples, we estimated the correlation between RNA concentration and PMI or RNA integrity and PMI. The RNA concentration was expressed in ng/µl and the integrity was inferred based on the RNA Integrity Number (RIN). We observed a similar degradation profile in all groups through the period studied, and we did not detect evidence of acceleration or retardation in the RNA decay influenced directly by our concealment simulations (Fig. 1). It is important to highlight that we used the same mass of input tissue for RNA extractions to avoid introducing differences in the total RNA quantity and quality outcomes.

Then, we carried out genome-wide expression analyses on brain samples using the tissue collected immediately after death as control and brains from mice differently concealed for 48-h as test. We chose the 48-h time point because it was the highest PMI with suitable RIN for the microarray reactions (optimal RIN > 7.0). Considering all comparisons, we found 2,103 genes differentially expressed (Supplementary Dataset File S1 and S2 online). To better understand the distribution of our samples within the groups based on gene expression patterns, it was performed hierarchical clustering analysis.

**Figure 2:**
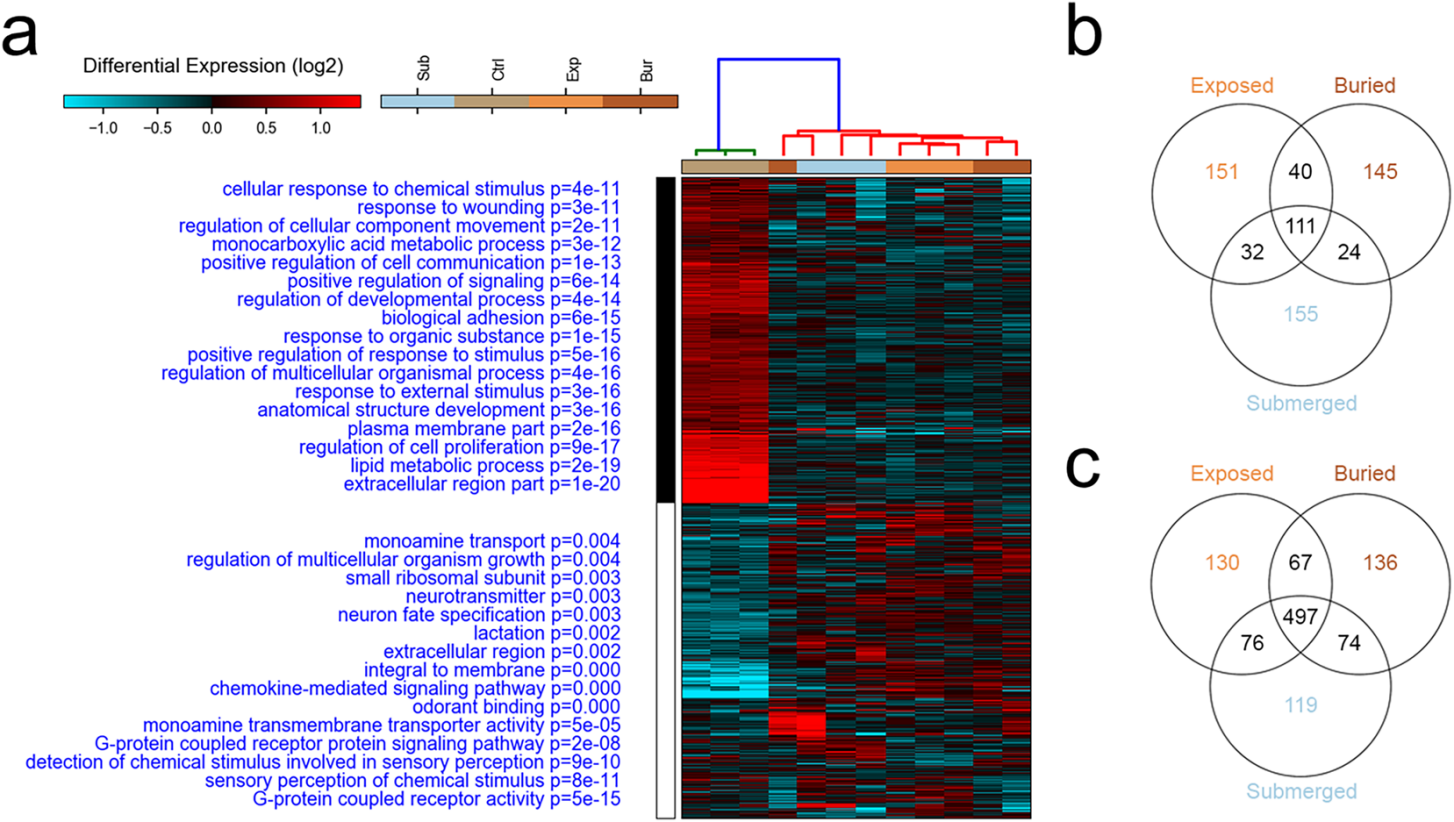
Post-mortem transcriptome in different concealment simulations. Hierarchical Clustering Analysis (**A**) indicates the separation of the controls (0 h PMI) from the concealment samples*.* Gene functions enriched for the differentially expressed genes and corresponding *P*-values are shown in blue on the left of the heatmap. Venn diagram of upregulated (**B**) and downregulated (**C**) genes show exclusive and common differentially expressed genes considering the concealment group Vs. control group. The list of genes described by Venn diagrams are included in Supplementary Dataset File S1 and S2 online). Concealment groups are indicated as Sub: Submerged; Ctrl: Control; Exp: Exposed; Bur: Buried.

Figure 2A shows that the main branches separate the control from the other groups, which are, in part, clustered into subgroups. Therefore, it can be argued that the variables ‘time’ and ‘environment’ may shape the post-mortem gene expression patterns. Interestingly, downregulated genes in concealed samples are prevalently involved in the active response to stimuli (*P*-values ranges between e-11 and e-17), in the regulation of metabolic processes (*P*-value = e-19), regulation of cell proliferation (*P*-value = e-17) and developmental processes (*P*value = e-14) (Fig. 2A). Upregulated genes are involved in the regulation of neuron fate, inflammation with chemokine signalling pathway, in the regulation of transcription by epigenetic mechanisms, and regulation of the translation with the synthesis of mRNAs for the small ribosomal subunits (Fig. 2A).

To perform the following analyses, we excluded the differentially expressed transcripts from comparisons that did not include the control group (Exposed Vs Submerged; Exposed Vs Buried; Submerged Vs Buried) because these ones do not contain information about the variable ‘time’. As described in Fig. 2B,C we identified 658 upregulated genes (Supplementary Dataset File S1 online) and 1099 downregulated genes in the concealed samples (Supplementary Dataset File S2 online). The downregulated genes exceeded those upregulated as resulted in other published works^6,20^. Figure 2B shows that 111 genes are upregulated in all considered conditions. Most of them are non-coding (47% snoRNA, 31% snRNA, 5% rRNA, and 2% miRNA), and 7% protein-coding; Supplementary Figure S1 and Supplementary Dataset File S1 online).

Searching for proteins encoded by commonly upregulated genes (7%) to potentially form complexes involved in specific functions, we evidenced that two of them (Atg16l1 and Rps20) participate in the formation of a regulatory network (Supplementary Figure S2) involved in the autophagy activation (Supplementary Figure S3). Similarly to what was done for upregulated genes, we analysed downregulated genes evidencing 497 commonly altered genes in all simulation groups when compared to the control (Fig. 2C). We highlighted that these genes encode for several interacting proteins (Supplementary Figure S4) prevalently involved in the metabolism regulation (Supplementary Figure S5).

### Biomarkers identification

Genome-wide gene expression analyses are useful tools for the identification of biomarkers for cell types or different conditions. Before the identification of biomarkers for the different coalescent conditions tested in this manuscript, we evaluated the goodness of fit of our data estimating the most likely cell types and tissues represented in our RNA samples. Expression profiles were comparable to those in the hippocampus, cerebral, prefrontal cortex, neurons, amygdala, cerebellum, oligodendrocytes, hypothalamus, olfactory bulb, and spinal cord (Supplementary Dataset File S3 online). All are components of the nervous system suggesting that no cell contaminations were introduced in our sample processing.

**Figure 3:**
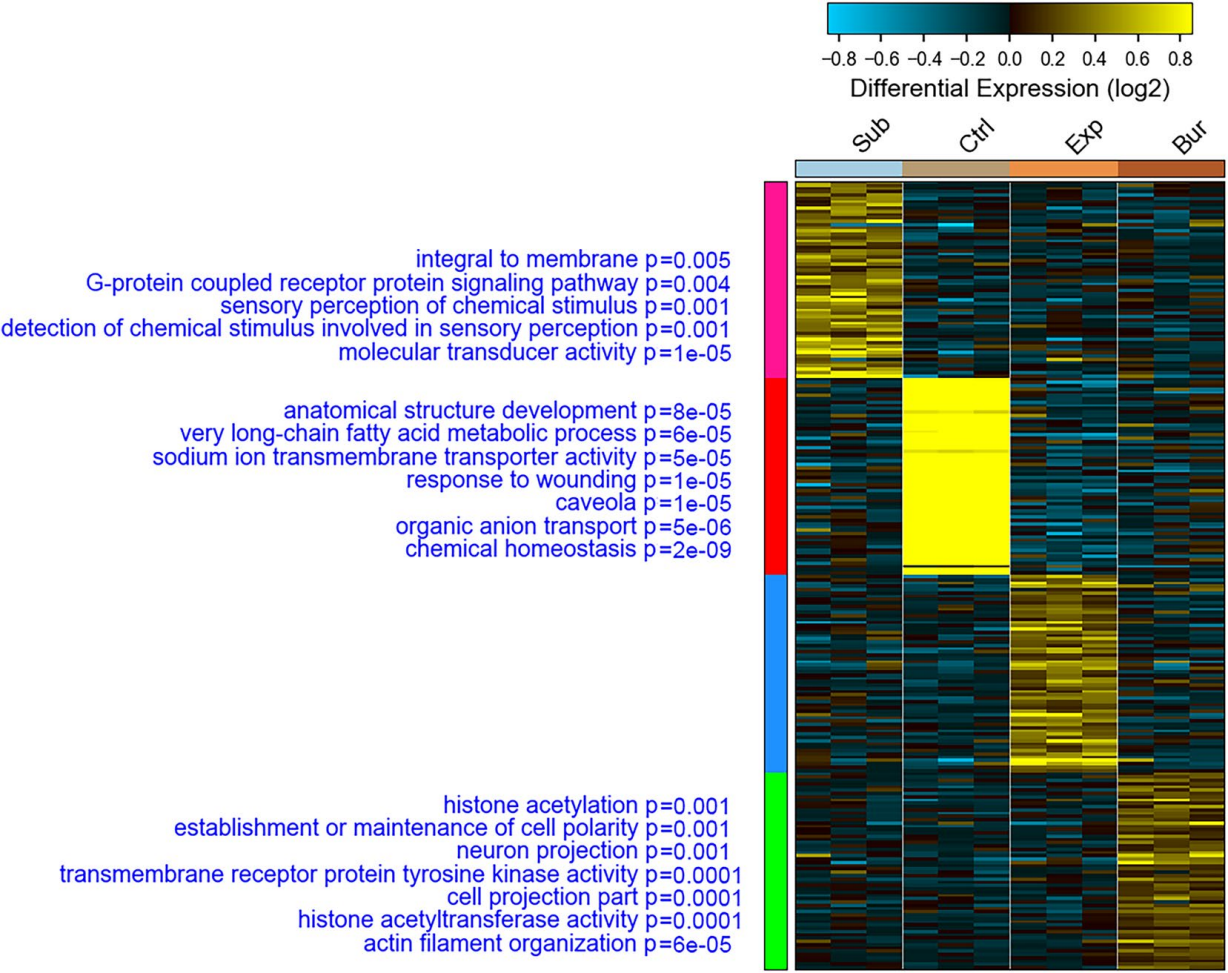
Biomarkers for each tested condition. Heatmap represents the expression of biomarkers identified using the AltAnalyzer algorithm. On the left of each biomarker group, indicated with the colours pink, red, blue, and green, are indicated enriched functional categories with the associated *P*-value.

We then identified biomarkers for each condition showing that only in biomarker genes for samples exposed to the air there were no particular enriched functions (Fig. 3 and Supplementary Dataset File S4 online). Instead, for the other conditions, biomarker genes were enriched in specific functions, mostly related to sensory perception in the submerged group and epigenetic modulation in the buried group (Fig. 3 and Supplementary Dataset File S4 online). Markers from the control group are those with the highest gene expression differences and are enriched for fatty acids metabolism, homeostasis, ion equilibrium, and response to wounding, all processes probably lost in the concealed samples. The preferential decay of RNA molecules was previously associated with the presence of AUUUA motif repeats within the 3’-UTR RNA sequence^21^. We evidenced that 63% of markers discriminate controls from all other treatments (red cluster in Fig. 3) contain AUUUA motif in their 3’-UTR (Supplementary Dataset File S5 online).

### Microarray gene expression validation

**Figure 4:**
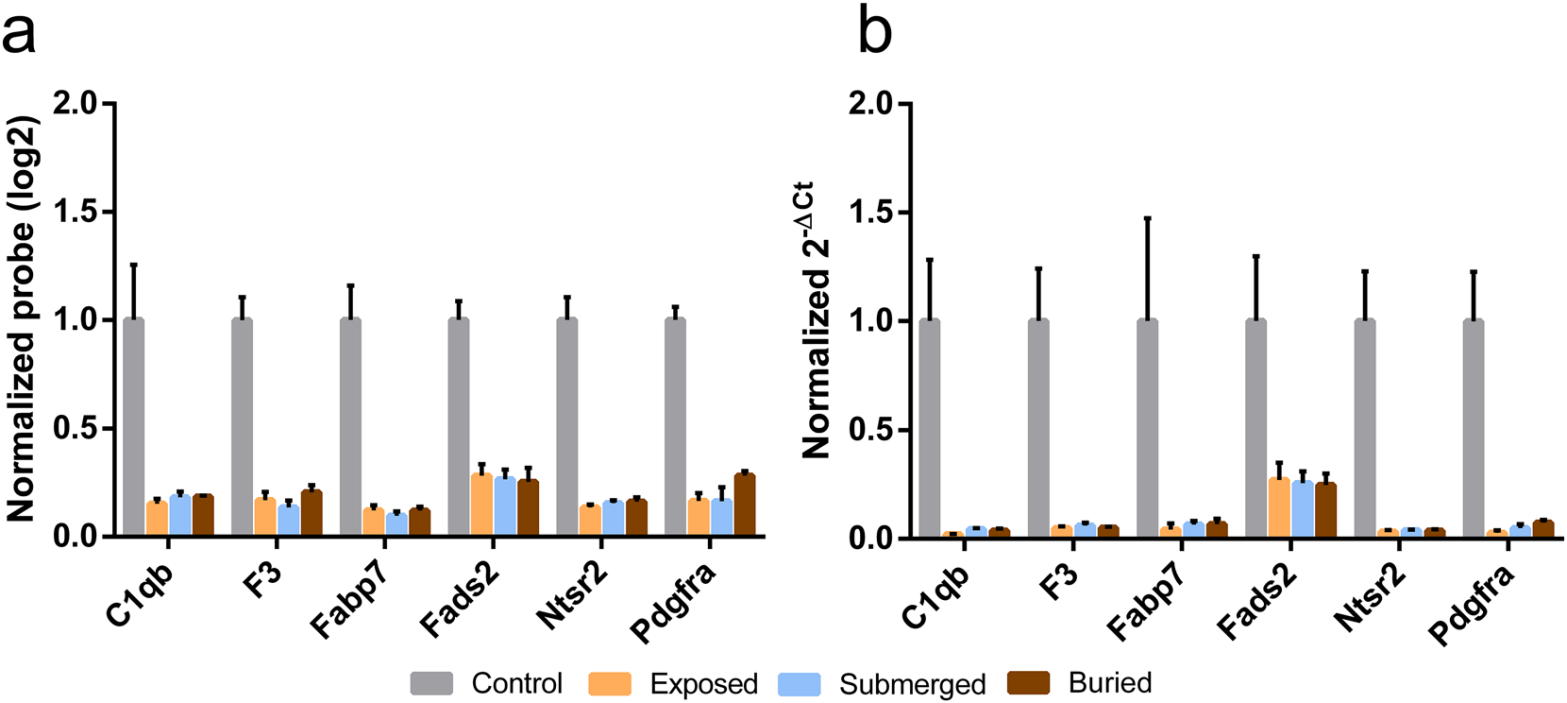
Microarray validation with RT-qPCR and selection of possible PMI markers. Gene expression of six genes from microarray experiments (**A**) were confirmed by RT-qPCR (**B**). For the RT-qPCRs we performed Multiple T-tests (Holm-Sidak method, *P* < 0.05) and all comparisons against controls were statistically significant. Control vs Exposed: C1qb (*P* = 0.0038), F3 (*P* = 0.0024), Fabp7 (*P* = 0.025), Fads2 (*P* = 0.015), Nts2 (*P* = 0.0018), Pdgfra (*P* = 0.0018). Control vs Submerged: C1qb (*P* = 0.0042), F3 (*P* = 0.0026), Fabp7 (*P* = 0.027), Fads2 (*P* = 0.013), Nts2 (*P* = 0.002), Pdgfra (*P* = 0.002). Control vs Buried: C1qb (*P* = 0.0041), F3 (*P* = 0.0025), Fabp7 (*P* = 0.027), Fads2 (*P* = 0.013), Nts2 (*P* = 0.0019), Pdgfra (*P* = 0.0021).

The real-time quantitative polymerase chain reaction (RT-qPCR) is an accurate and sensitive method to detect variations in RNA levels (through the amplification of the retrotranscribed RNA) commonly used as a validation tool for confirming gene expression results obtained from microarray analysis. For the validation experiments, we chose genes downregulated in all concealment simulations, with a known function, and that have a homologous in the human genome. Figure 4 shows the expression both calculated with microarray and RT-qPCR experiments of the selected genes. Correlations between microarray and RT-qPCR gene expressions are very close to 1 (C1qb = 0.92, F3 = 0.97, Fabp7 = 0.96, Fads2 = 0.94, Ntsr2 = 0.99, Pdgfra = 0.95) making us confident on the validity and reproducibility of the microarray results.

The complement component 1, q subcomponent, beta polypeptide gene (C1qb), the fatty acid binding protein 7 (Fabp7), and the neurotensin receptor 2 (Ntsr2) genes are included in the list of biomarkers for the red cluster described in Fig. 3 (Supplementary Dataset File S4 online), while the coagulation factor III (F3), the fatty acid desaturase 2 (Fads2), and the platelet derived growth factor receptor, alpha polypeptide (Pdgfra) genes are downregulated in all tested conditions (Supplementary Dataset File S2 online). C1qb gene encodes the B-chain of a polypeptide involved in the complement cascade, which is part of the innate immune system^22^ and the F3 gene encodes a cell surface glycoprotein involved in blood coagulation cascades^23^. Altogether, these two genes are components of the gene sets ‘response to wounding’, ‘extracellular region part’ (C1qb) and ‘plasma membrane part’ (F3) (Fig. 2A). Fabp7 and Fads2 genes are associated with the ‘lipid metabolic process’ and ‘regulation of developmental process’. The Fabp7 protein binds long-chain fatty acids and plays a role during brain development^24^. Fads2 is a fatty acid desaturase involved in the biosynthesis of highly unsaturated fatty acids important for nervous system function^25^. Ntsr2 gene codifies for a neurotensin receptor expressed on the brain involved in thermal perception^26^ (‘response to external stimulus’) while the Pdgfra gene codifies for a growth factor for cells of mesenchymal origin^27^ (‘anatomical structure development’).

To evaluate the applicability of this study also in human samples, we considered the expression of these genes in different human tissues retrieving data from the Genotype-Tissue Expression (GTEx) Project. We were interested in understanding if all genes considered for the RT-qPCR validations were expressed in the human brain. We evidenced that all genes selected are expressed in the human brain (Supplementary Figures S6–S11). PDGFRA shows the lower expression, especially in the cerebellum. Interestingly, the cerebellum is the organ that shows the lower expression for all the considered genes but the FABP7, which is most expressed in this area. Considering the expression profile in human samples, NTSR2 and FABP7 may be considered brain specific genes.

## Discussion

Once we understand the cellular processes that occur after the organismal death, we are closer to understanding life itself. Additionally, several applications can be established with the thanatotranscriptome analysis—which is the set of transcripts that are detected after death. This field of study can be explored in forensic sciences (e.g. determination of the cause of death; estimation of post-mortem interval; identification of body fluids)^13^ and in medical sciences (e.g. quality control for organ transplantation)^2,18^. Previous studies investigated the dynamics of gene expression after death in human liver samples^28^, human prostate tissue^29^, zebrafish and mice brains^6,30^ and 7105 human samples from 36 tissues recovered from the GTEx project, concluding that the brain (cortex and cerebellum) and spleen were the tissues presenting the higher RNA stability^18^. This can be explained because the brain is protected by the neurocranium, thus being less exposed to exogenous RNases^20^. However, a major drawback of these studies is that they were performed under controlled conditions, which restricts the application of real-life practice. Here, we analysed brains from mice recovered from different concealment simulations, to evaluate environmental influences in the post-mortem transcript abundance.

Since bodies have different decomposition processes depending on the environment in which they are deposited^31,32^, different rates of RNA degradation can be expected. Previous works demonstrated that temperature is a factor that acts directly on RNA degradation^16,33^. The degradation profile similarity (Fig. 1) could partially be explained by the temperature and room which were controlled. However, even with controlled conditions and non-statistical differences in total RNA degradation rate, we detected distinct transcriptional profiles associated with each environment tested, showing that the environment can shape the thanatotranscriptome (Fig. 2).

Most of the upregulated genes detected in our analysis were non-coding RNAs (Supplementary Figure S1 and Supplementary Dataset File S1 online). Small nucleolar RNAs (snoRNAs) are a class of RNA that primarily guide chemical modifications of other RNAs such as ribosomal (rRNAs), transfer (tRNAs), and small nuclear RNAs (snRNAs)^34^. Moreover, they are also involved in the RNA splicing regulation as snRNAs. rRNAs and tRNAs are involved in protein synthesis. Differently, miRNAs exert their function hybridizing to mRNA 3’ untranslated region (3-UTR). The miRNA-mRNA pairing induces both the inhibition of mRNA translation and its degradation^35,36^. These alterations evidence the modification of the RNA homeostasis during the post-mortem period may compromise cell homeostasis. Moreover, our data showed that Atg16l1 and Rps20, involved in autophagy activation, were upregulated genes 48 h after death (Supplementary Figures S2 and S3 online). This may reflect the activation of a specific intracellular degradative process through lysosomes with Atg16l1 that has a central role in the regulation of the autophagy network^37^. Autophagy is implicated in the pathogenesis of major neurodegenerative disorders^38^ because it is involved in the protection against the formation of neuroaggregates^39^ and its activation appears as an extreme response to counteract processes involved with ageing and organismal death.

Pozhitkov et al.^2^ also analysed mRNAs actively transcribed after death and they found 515 upregulated genes, including genes involved with epigenetic regulation and inflammation pathways that are in parallel with our assays. We identified a comparable number of upregulated genes in our concealed samples (Supplementary Dataset File S1 online). Even though it seems reasonable to think that the described alterations might occur, it is important to keep in mind that these processes can be triggered by active or stochastic mechanisms. However, regardless of the cause of the gene expression alteration, the outcome must influence the previously described functions. In contrast, Catts et al. analysed seven time-points after death (0, 6, 12, 18, 24, 36, 48 h) and reported 1044 downregulated genes from post-mortem brains of mice identifying three major pathways significantly downregulated: regulation of nucleic acid metabolism; regulation of transcription; and regulation of transcription DNA-dependent^6^. We detected a comparable number of downregulated genes considering the 48-h samples of all three concealment groups with functional enrichments comparable with those described by Catts et al. and with an average downregulation of 50% in all concealment groups (Supplementary Dataset File S2 online). These downregulated genes were mostly related to metabolism regulation (Supplementary Figure S5 online), which is in agreement with the decrease of oxidative metabolism after death in response to the diminished circulating oxygen^40^.

Our genome-wide experiments do not allow the evaluation of RNA degradation, therefore the marked downregulation we evidenced may be attributed to the combination of a reduced RNA synthesis and/or to the RNA degradation. Interestingly, Catts et al. associated the preferential decay of RNA molecules containing the AUUUA motif repeats, which can be involved in the recruitment of RNA-binding factors that facilitate the mRNA degradation^6,21^. We showed that 63% of the markers in our analysis contain AUUUA motif in their 3’-UTR (Supplementary Dataset File S5 online). This result supports the concept that the downregulation in our tested samples may be due to mechanisms that influence the half-life of RNA molecules. Altogether, these data suggest that death and the timing of shutting down an organism, an organ, a tissue and a cell is a gradual, dynamic and coordinated process. A live cell from a dead organism might even try to synthesize proteins to re-establish homeostasis, considering that ‘small ribosome unit’ and ‘regulation of multicellular organism growth’ are upregulated gene functions after death (Fig. 2).

An important issue is that the transcriptome can respond to environmental signals and the interaction of the dead organism with the surrounding environment should be considered in the search for molecular markers as forensic tools. Markers for buried samples are enriched for epigenetic gene expression, and modulation and neuronal projection regulation (Fig. 3). Epigenetic modulation can occur in response to environmental stimuli, such as physicochemical factors (temperature, pH) or biological factors (microbiota) present in the soil. Markers for the submerged samples are enriched for the function of transducing sensory stimuli (Fig. 3). Animals from all groups were euthanized using the same method (CO_2_ exposure), which could stimulate sensorial genes. However, we observed the upregulation of sensorial genes that also are part of biomarkers for this condition only in the submerged group. For this reason, we hypothesized that the water that infiltrated the respiratory tract and the oral cavity can interact with cells involved in the sensory tests of the ambient. Thus, even in the post-mortem period, these cells could send sensory signals to the brain cells, which synthesize mRNAs involved in the perception of these senses.

Death is a multi-step and gradual event. After an organism dies, the remaining living cells may try to overcome this event, by producing molecules that would allow an extended cellular functioning, activating the transcription by epigenetic mechanisms and processes involved in the RNA homeostasis. Clearly, cells do not immediately cease their function when the body shuts down. Our data show that RNA degradation after death is a progressive event and that a considerable number of upregulated genes can be found even two days after clinical death. Furthermore, as far as we know, this is the first work that investigates post-mortem transcriptome considering body concealment of mice. We identified different gene markers for each tested condition that can be used in future studies to estimate the post-mortem period considering the surrounding environment. In summary, our work: (i) shows that some genes respond to specific environmental signals and some transcripts have a similar degradation or upregulation pattern after death, regardless of the surrounding environment; (ii) proposes a list of molecular markers for each concealment condition studied or independent from the concealment conditions and (iii) paves the way for further discussion about cell activity and molecular pathways altered after death opening new horizons in forensic practice.

## Methods

### Ethics statement

The protocol used in this study was approved by The Ethical Committee on Animal Use (CEUA) of University of Brasilia (UnBDOC 79,518/2013). All experiments were performed in accordance with relevant guidelines and regulations. All procedures were made according to the ARRIVE guidelines (PLoS Bio 8(6), e1000412,2010​).

### Animals

A total of 48 healthy male mice (*Mus musculus*) of the C57BL/6jn lineage, aged 16 weeks and with similar weight were used for this experiment. The animals were housed in polypropylene cages and maintained under controlled temperature, humidity and a light/dark 12 h/12 h cycle with free access to commercial food and filtered water ad libitum*.* Animals were fasted for eight hours prior to the experiments in order to minimize the impact of food ingested on body degradation. All animals were euthanized by CO_2_ exposure at the same time. To investigate the impact of different environments on gene expression, three mice corpses were used as control (time 0 h) and 45 distributed in three experimental groups of 15 bodies each: i. exposed group; ii. buried group and iii. submerged group. To mimic the proposed microenvironments, each mouse carcass was placed in individual sealed plastic bags (High Density Polyethylene), as follows: i. exposed group: simulation of bodies exposed to environmental air using sealed plastic bag compartment without the extraction of the surrounding air; ii. buried group: simulation of burial concealment using sealed plastic bags with autoclaved soil. The soil samples were performed with a hand trowel up to 10 cm depth at University of Brasília *Campus*. The soil was classified as Ferralsols according to World Reference Base Soil Resources^41^. These soils occur mainly under tropical climates and considered as being strongly weathered associated with old geomorphic surfaces (IUSS-WRB, 2015). In Brazil, ferralsols are clayey to very clayey, dystrophic and acidic, with pH between 4.0 e 5.5^42^; iii. submerged group: simulation of submersion concealment using sealed plastic bags with Milli-Q autoclaved water (pH 6.8). The experiments were carried out in a room with controlled temperature (23 ºC) and humidity (55%). The room temperature and humidity were measured daily by a digital thermo-hygrometer (TFA Dostmann, Germany). To analyse gene expression, whole brain of three animals of each group was collected over five time periods (24, 48, 72, 96 and 192 h), stored in 1.5 ml tubes, immediately frozen in liquid nitrogen. For the control group, brains from three animals without simulation of cadaver concealment were extracted, immediately after death (time 0). Tissue samples were kept at -80 ºC until RNA extraction. To minimize the degradation by RNases present in the laboratory environment, all materials and surfaces used for animal dissection and mRNAs extraction were treated with 1% diethylpyrocarbonate (DEPC) (Thermo Fisher Scientific).

### Extraction and quantification of RNA

The brain tissue was homogenized and macerated in liquid nitrogen and a 30 mg aliquot was used for RNA extraction using the Illustra RNAspin Mini Isolation Kit (GE Healthcare) according to the manufacturer's guidelines. The concentration of the extracted RNA was quantified with NanoDrop 2000 (Thermo Fisher Scientific) and RNA integrity was tested with the 2100 Bioanalyzer (Agilent Technologies). To detect if the RNA concentration (ng/µl) and integrity (RNA Integrity Number—RIN) are different between groups we performed a linear regression analysis and compared the slopes of each condition tested. The RIN is a number that is calculated using the Agilent Bioanalyzer and that is directly related to RNA degradation. We confirmed the statistical significance by ANOVA (Tukey correction method, *P* < 0.05) using the average values of a concealment group in comparison with the other groups.

### Microarray analysis

To analyse the mouse brain transcriptome, we used the Affymetrix GeneChip MoGene 2.0 ST Array (Applied Biosystems), according to the manufacturer's protocol. We used 100 ng of RNA as input for all samples, control 0 h, exposed 48 h, buried 48 h and submerged 48 h, each in three biological replicates. Microarray data were normalized with the R suite using the RMA normalization. Data are submitted in the GEO database to allow independent analyses (GSE160147)^43^. The identification of differentially expressed genes, enrichment and cluster analyses were performed with the AltAnalyze platform^44^. Searching analysis for the most likely cell types and tissues represented in our RNA samples was performed using the lineage profile analysis implemented in the AltAnalyze platform. Microarray gene expression was also used to identify specific markers for each condition (Marker-Finder in the AltAnalyzer platform). Parameters used for the identification of differentially expressed genes are the following: genes expressed below 1 were filtered out, comparison groups with moderate t-test with *P*-value corrected with Benjamini–Hochberg lower than 0.05. Gene enrichment analyses were performed on genes having 1.5 as the minimum gene expression fold change and 0.05 as the maximum *P*-value for t-tests. Gene enrichment parameters were: z-score cut off: 1.96; *P*-value based on 2000 permutations lower than 0.05; minimum number of genes in a gene set: 3. To identify interlinked genes and construct functional networks we used the network topology-based analysis with the WEB-based Gene SeT AnaLysis Toolkit^45^ using default parameters. Networks were constructed based on the protein–protein interaction database collected in Biogrid^46^. AUUUA motifs search in the 3’-UTRs was performed with the AREsite2 using default parameters^47^.

### RT-qPCR

To validate the microarray results, we carried out RT-qPCR gene expression analysis of six mRNAs that were downregulated in all experimental situations (48 h post-mortem) compared with the control sample. cDNA molecules from each reaction were obtained from 10 ng of total RNA with the High-Capacity RNA-to-cDNA kit (Applied Biosystems), following the manufacturer's instructions. The amplification reactions were carried out in the Step-One Plus Real-Time PCR System (Applied Biosystems) with the SYBR® Green Master Mix (Applied Biosystems). All primers were validated with a standard curve with 100% (± 10%) efficiency. Technical triplicates of each biological sample of the experiment were performed. The primer sequences are listed in Supplementary Dataset File S6 online, including the Actb, used as the reference gene. For detecting statistical significance in RT-qPCR experiments performed for microarray validation, we used Multiple T-tests (Holm-Sidak method, *P* < 0.05). To evaluate the similarity of gene profiles derived from microarray and RT-qPCR gene expression analyses, the Pearson correlation among all the 12 normalized samples (3 controls, 3 exposed, 3 submerged, 3 buried) was used.

## Supplementary Information

Supplementary Information 1.

Supplementary Information 2.

Supplementary Information 3.

Supplementary Information 4.

Supplementary Information 5.

Supplementary Information 6.

Supplementary Information 7.
