# Supplementary material for: Insights into how environment shapes post-mortem RNA transcription in mouse brain
Source: Sci Rep. 2021 Jun 21;11:13008. doi: 10.1038/s41598-021-92268-y (PMC8217559; doi:10.1038/s41598-021-92268-y)

Insights into how environment shapes *post-mortem* RNA transcription in the brain of mouse

Raphael Severino Bonadio [1,3]; Larissa Barbosa Nunes[1]; Patricia Natália S. Moretti [2]; Juliana Forte Mazzeu[2], Stefano Cagnin [3], Aline Pic-Taylor [1], Silviene Fabiana de Oliveira [1*]

[1] Department of Genetics and Morphology, University of Brasilia, Brasilia, Brazil.
[2] Faculty of Medicine, University of Brasilia, Brasilia, Brazil.
[3] Department of Biology and CRIBI Biotechnology Centre, University of Padova, Padova, Italy.

*e-mail: silviene.oliveira@gmail.com

Supplementary Figure S1. Categorization of commonly upregulated genes considering the comparisons control vs buried, control vs exposed, and control vs submerged samples.

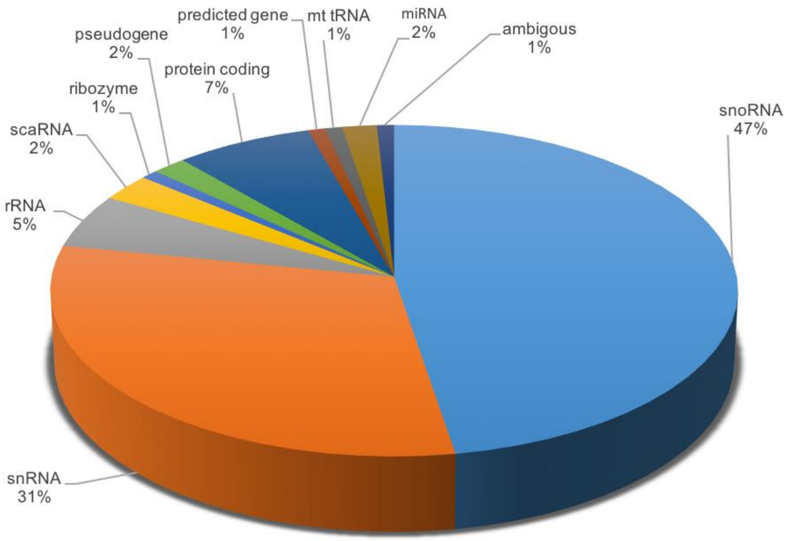

Supplementary Figure S2. Network of interacting proteins encoded from commonly upregulated genes in all concealment groups. In red are evidenced proteins involved in the macro autophagy. Bigger nodes in the networks are the seeds while the smaller ones are the top-ranking neighbours. Seeds represent differentially expressed genes.

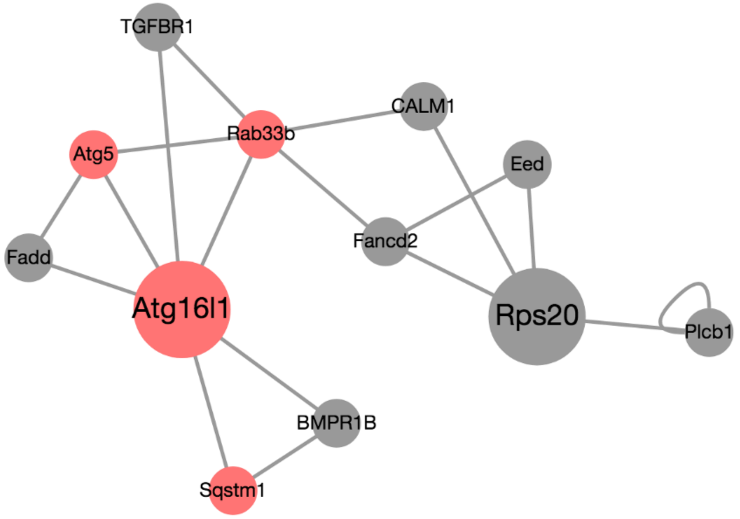

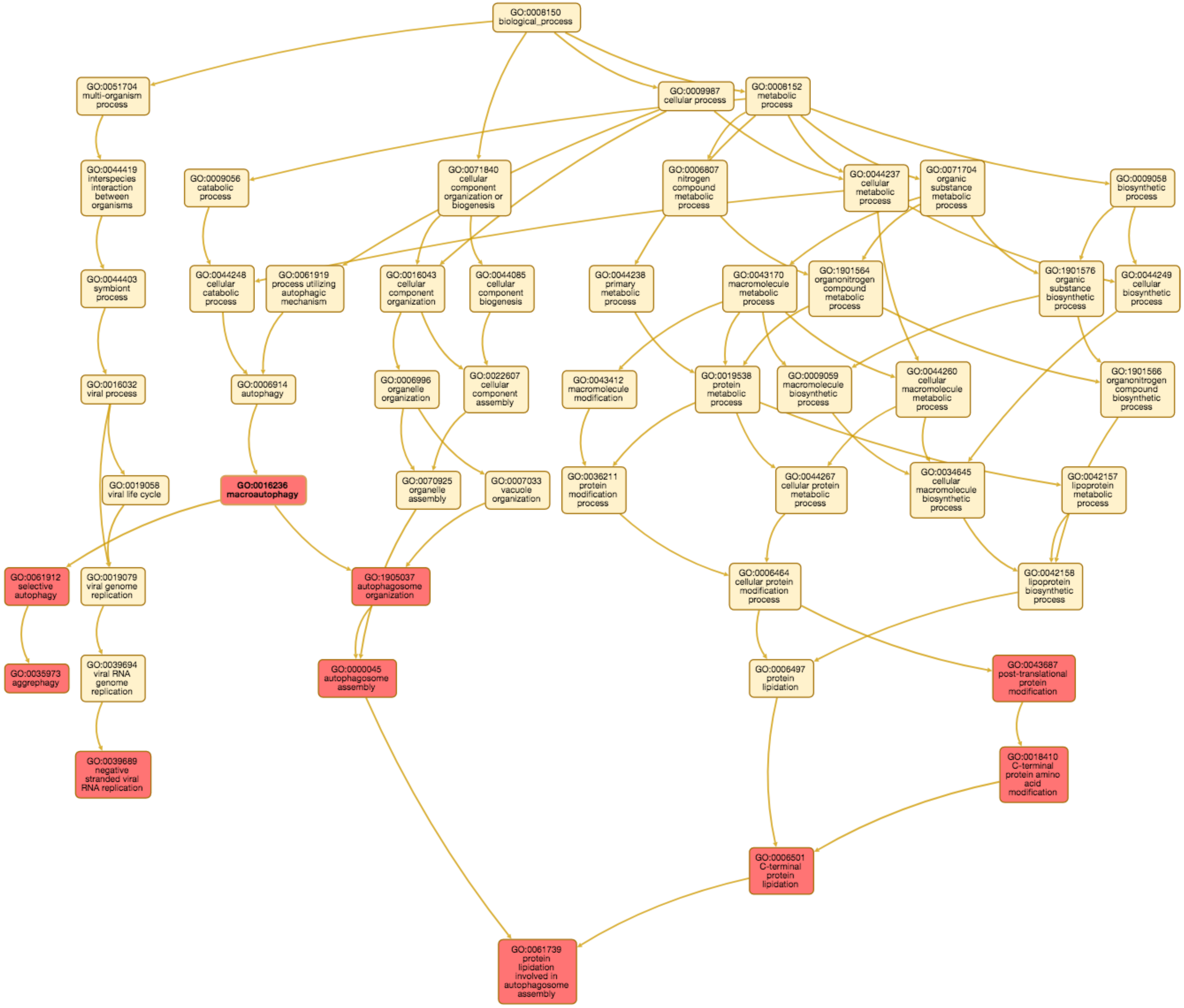

Supplementary Figure S3. Network of enriched biological processes from commonly upregulated genes in all concealment groups. Dark red squares indicate high statistical power (p-value<10^{-3}) and yellow squares indicate low statistical power (p-value>0.05). Altogether, the enriched processes are involved in post-translational protein modifications involved in autophagy.

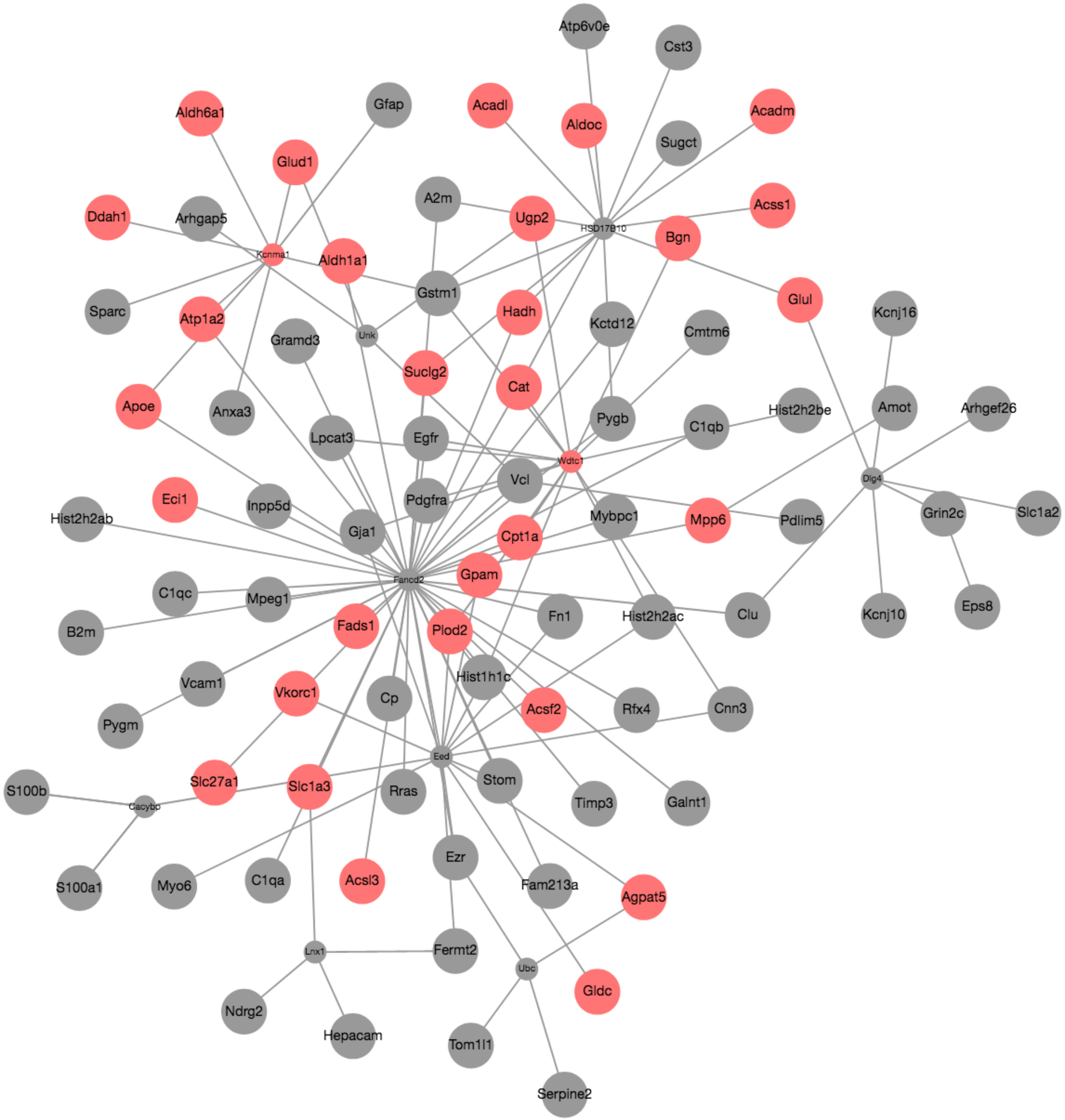

Supplementary Figure S4. Network of interacting proteins encoded from commonly downregulated genes in all concealment groups. In red are evidenced proteins involved in the small molecule metabolic process. Bigger nodes in the networks are the seeds while the smaller ones are the top-ranking neighbours. Seeds represent differentially expressed genes.

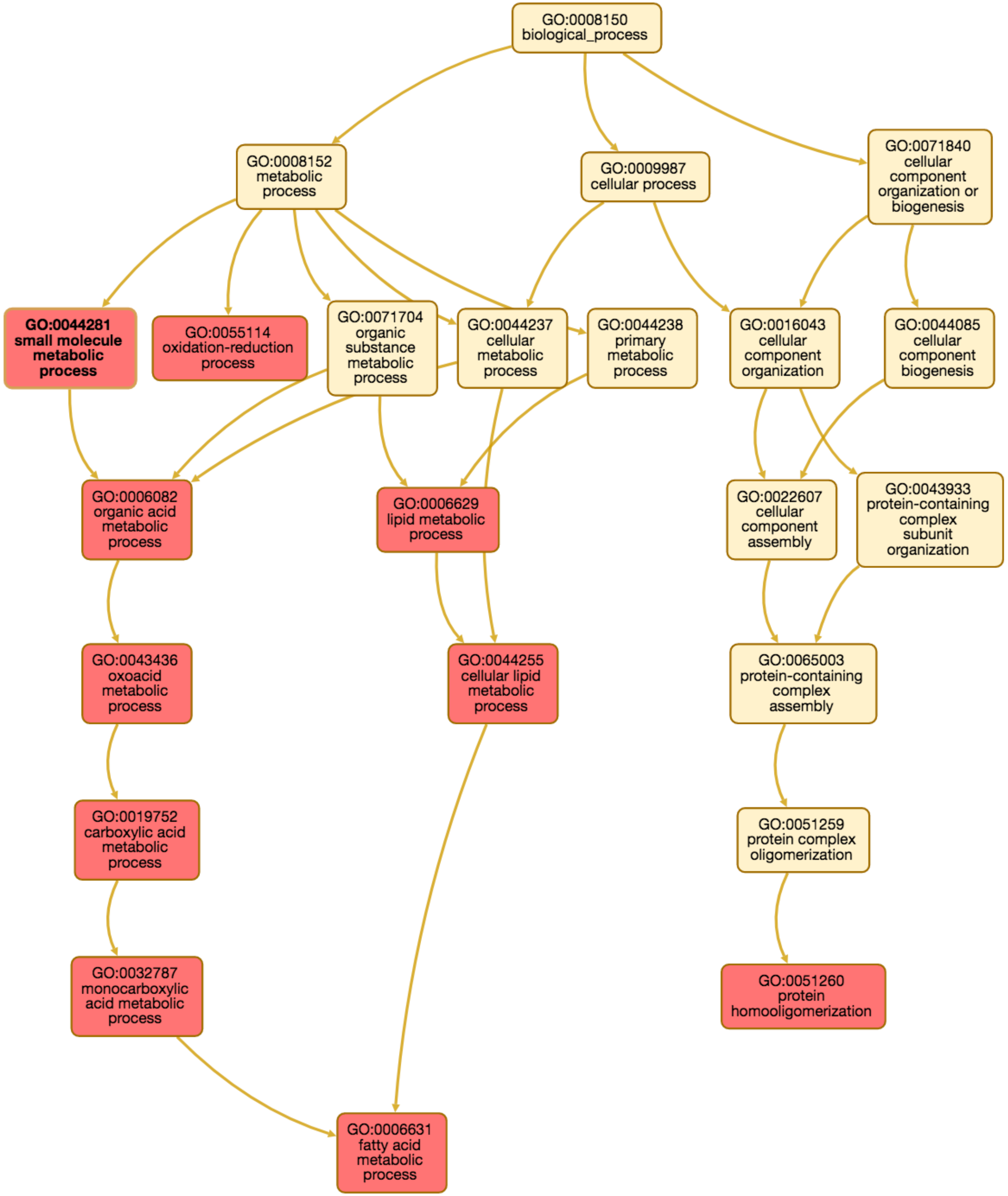

Supplementary Figure S5. Network of enriched biological processes from commonly downregulated genes in all concealment groups. Dark red squares indicate high statistical power (p-value<10^{-3}) and yellow squares indicate low statistical power (p-value>0.05). Altogether, the enriched processes are involved in small molecule and fatty acid metabolic processes.

Supplementary Figure S6. Expression of C1QB in different human samples. The expression is in Transcripts Per Million (TPM). It means that should be read as for every 1,000,000 RNA molecules in the RNA-seq sample.

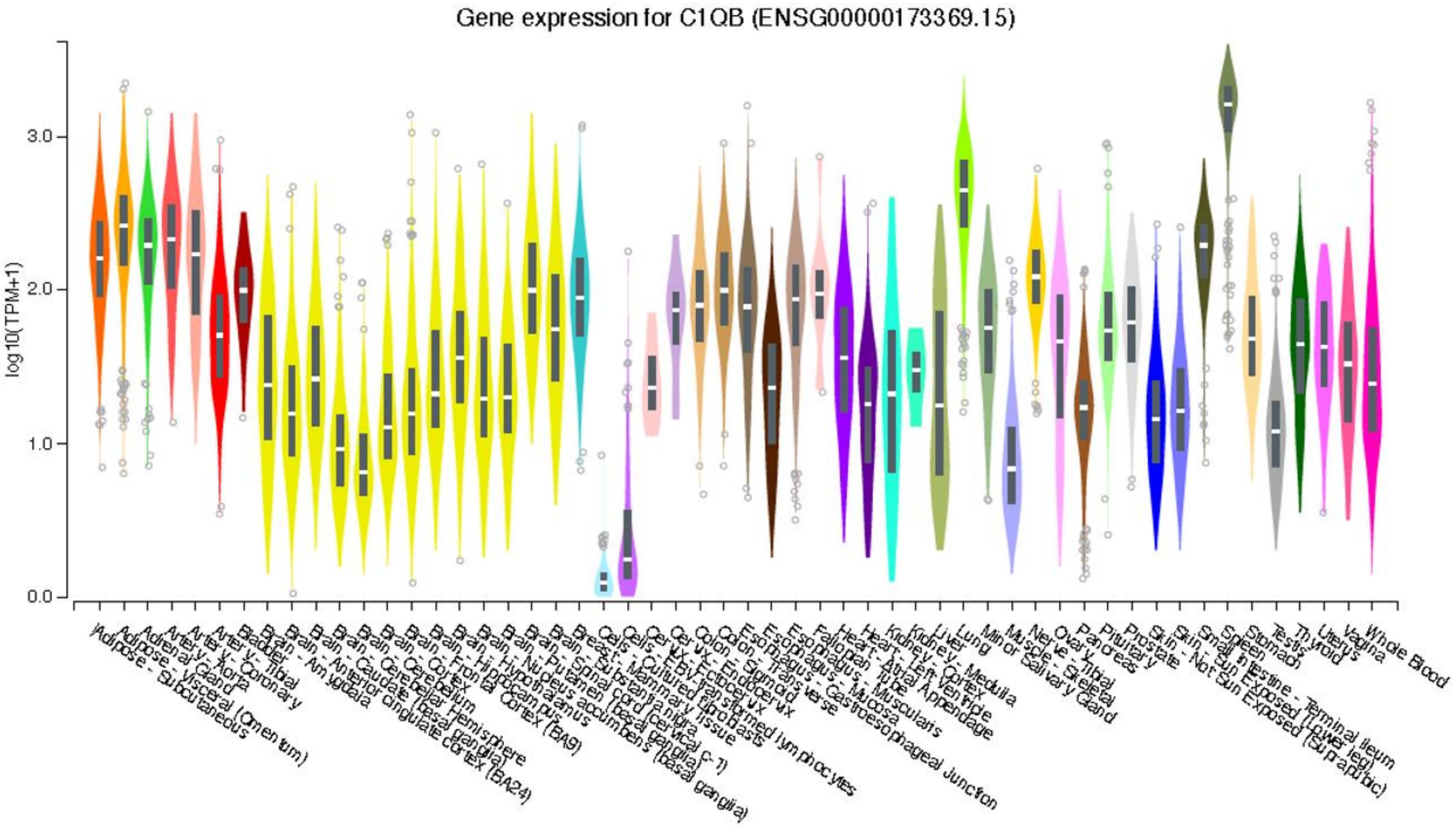

Supplementary Figure S7. Expression of FABP7 in different human samples. The expression is in Transcripts Per Million (TPM). It means that should be read as for every 1,000,000 RNA molecules in the RNA-seq sample.

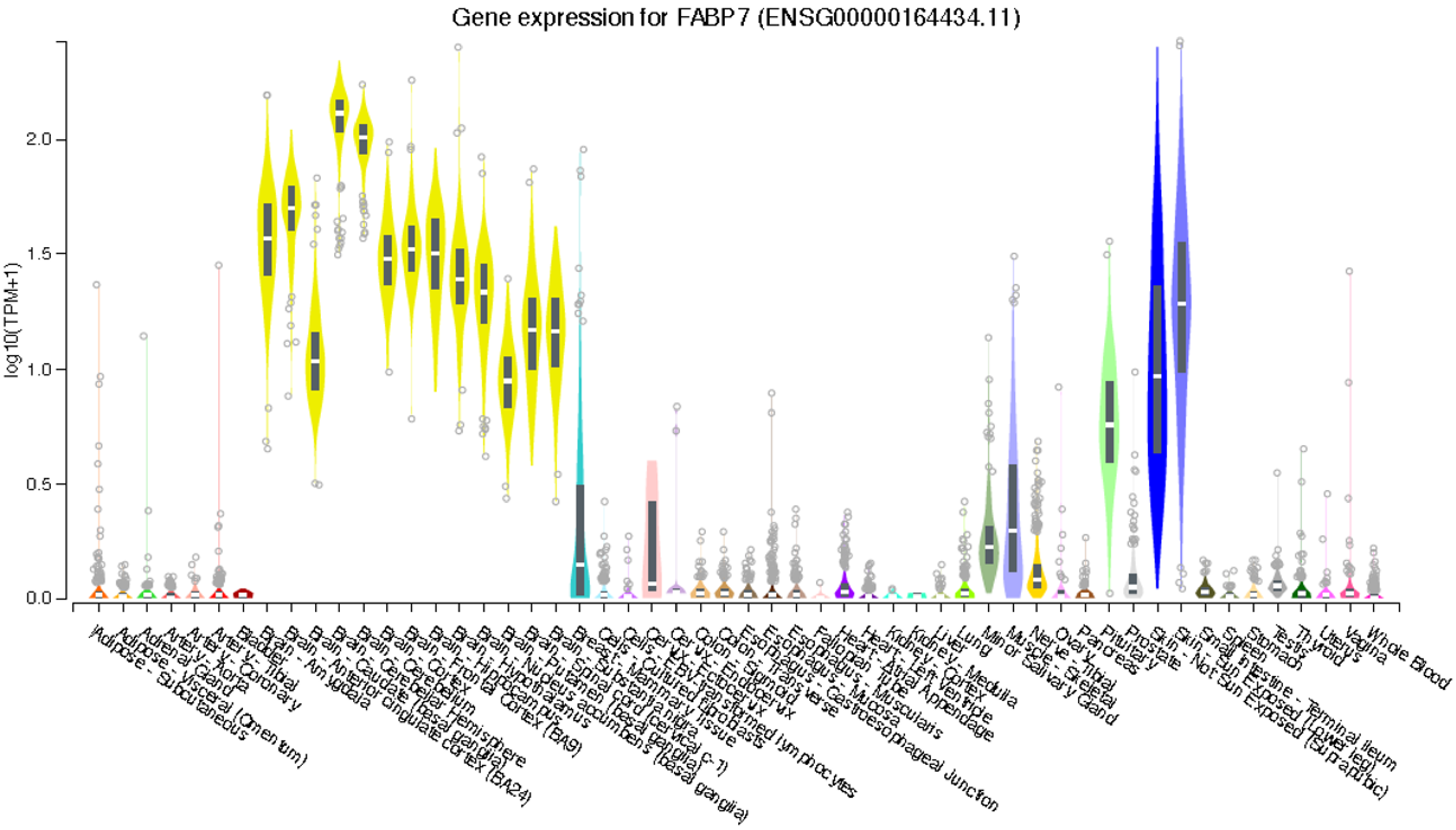

Supplementary Figure S8. Expression of NTSR2 in different human samples. The expression is in Transcripts Per Million (TPM). It means that should be read as for every 1,000,000 RNA molecules in the RNA-seq sample.

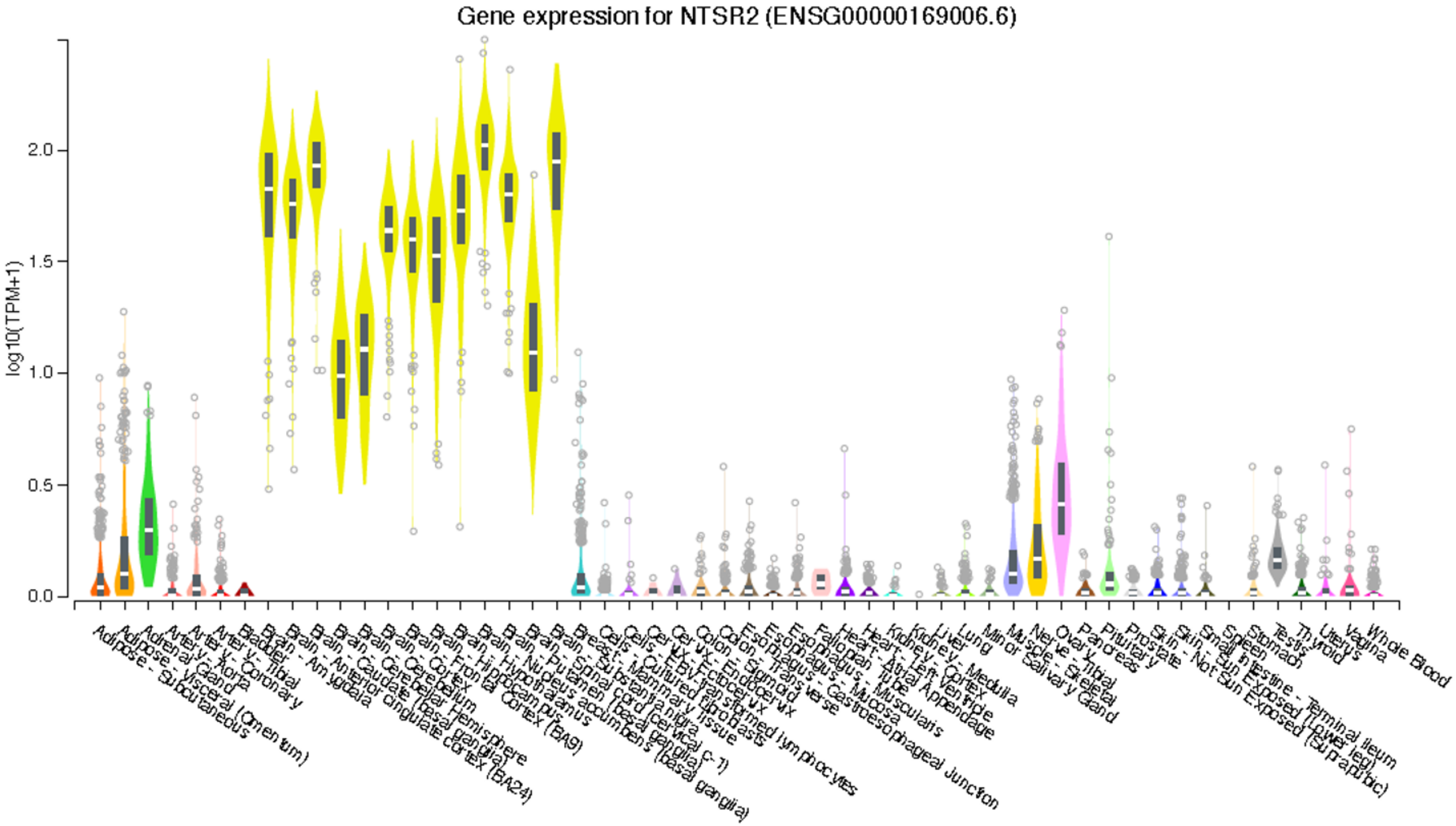

Supplementary Figure S9. Expression of F3 in different human samples. The expression is in Transcripts Per Million (TPM). It means that should be read as for every 1,000,000 RNA molecules in the RNA-seq sample.

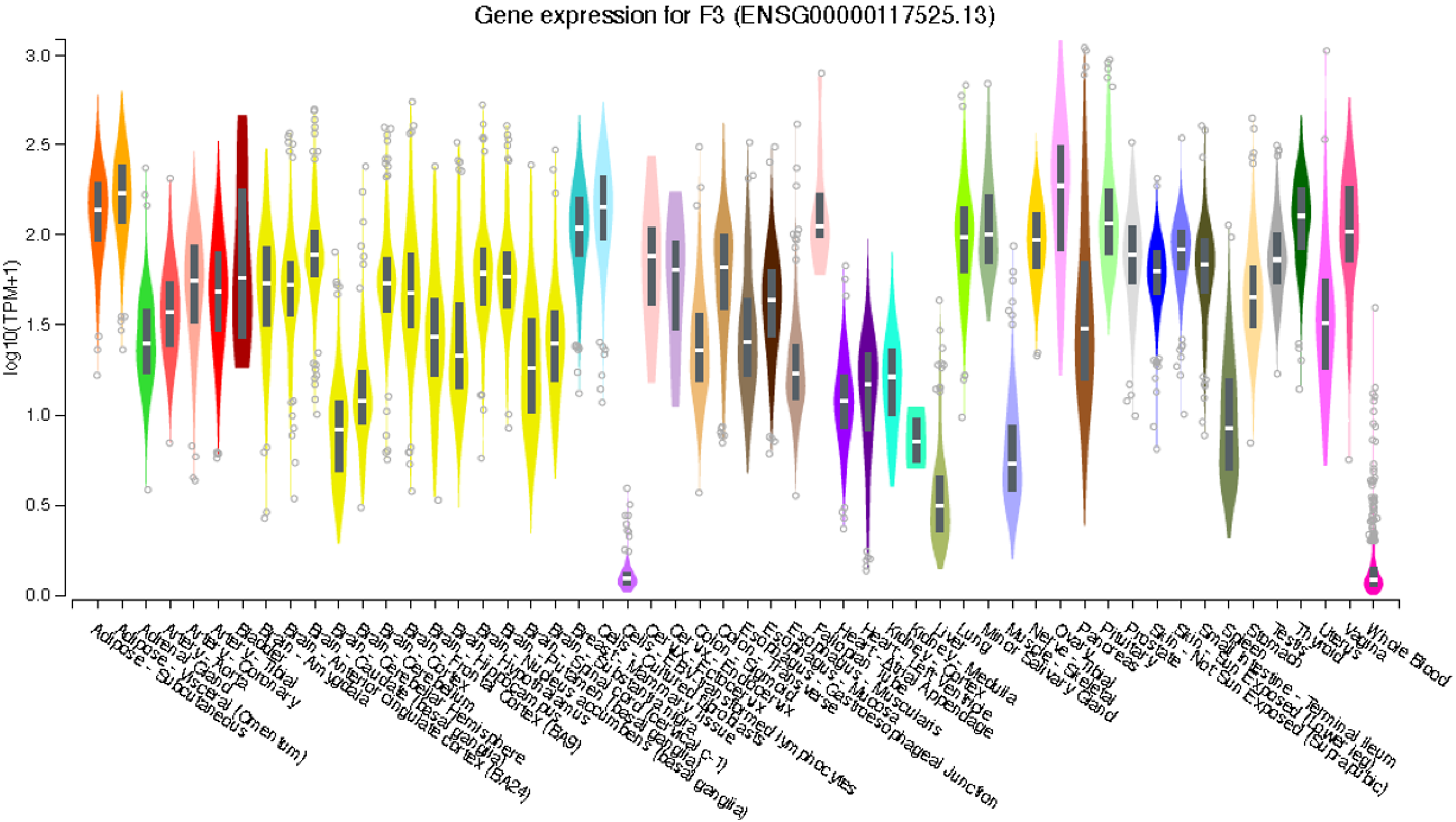

Supplementary Figure S10. Expression of FADS2 in different human samples. The expression is in Transcripts Per Million (TPM). It means that should be read as for every 1,000,000 RNA molecules in the RNA-seq sample.

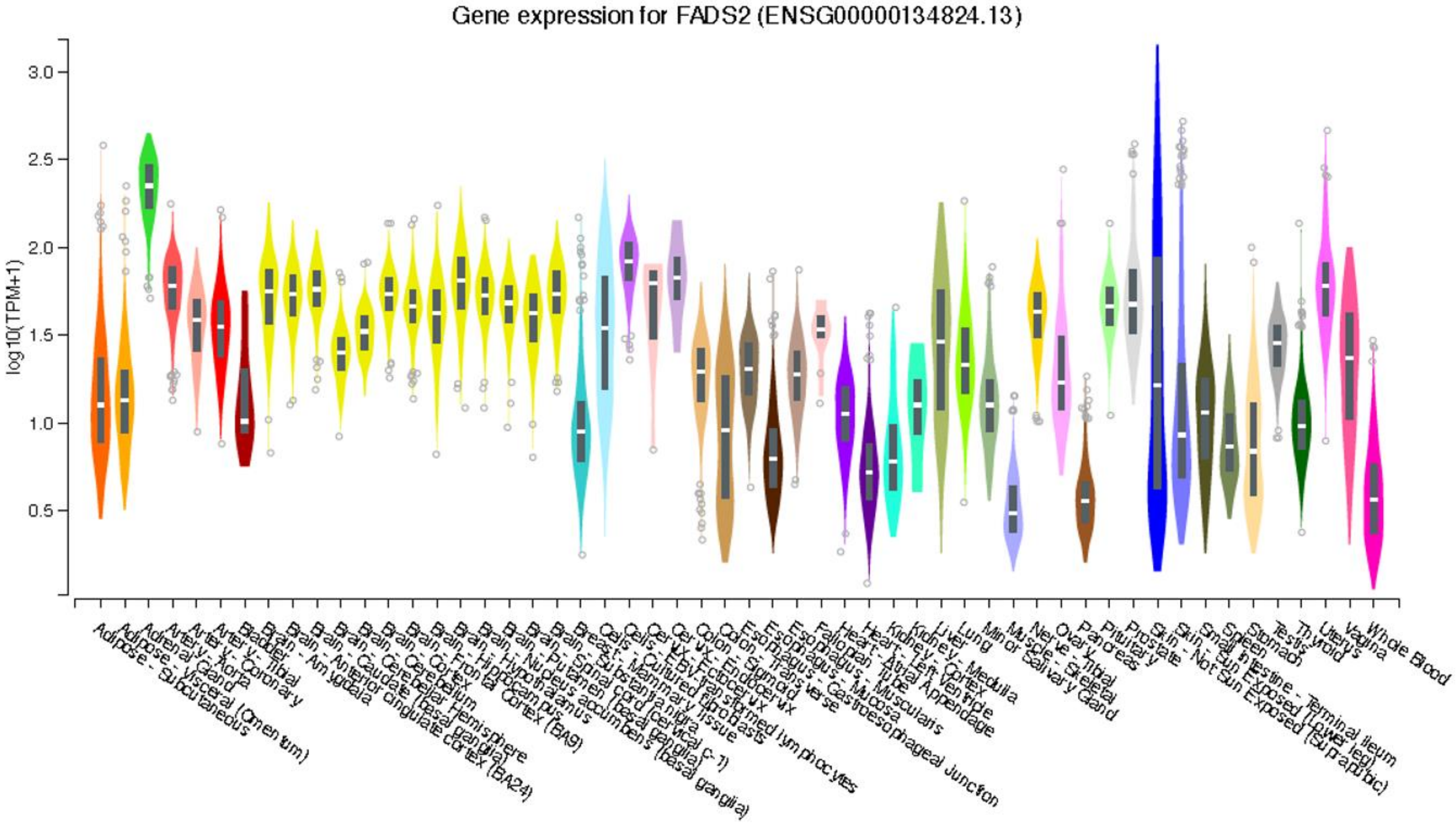

Supplementary Figure S11. Expression of PDGFRA in different human samples. The expression is in Transcripts Per Million (TPM). It means that should be read as for every 1,000,000 RNA molecules in the RNA-seq sample.

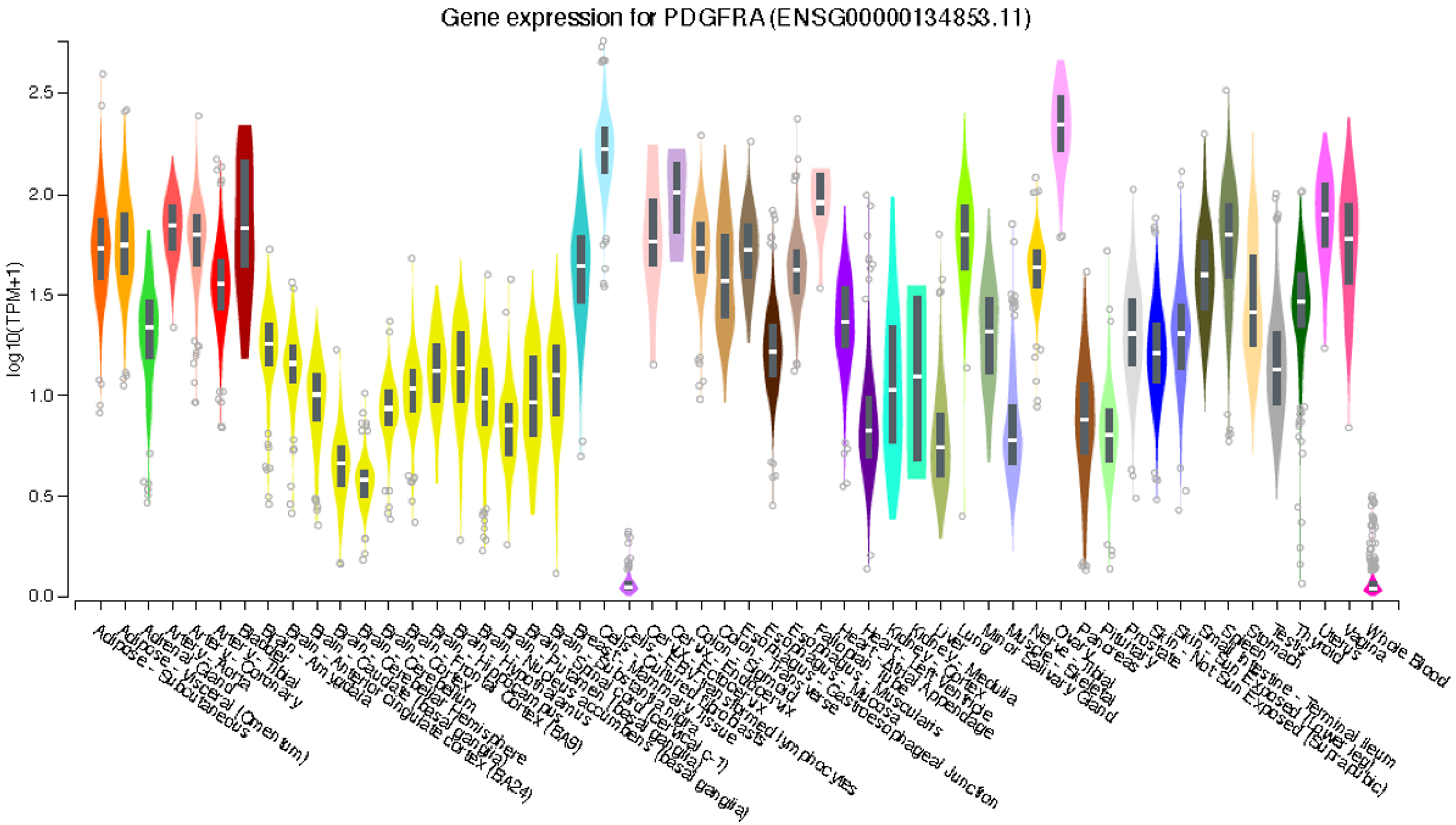

Supplement: Supplementary file 7 — Supplementary Information 7. [file 41598_2021_92268_MOESM7_ESM.pdf]
